# Long-term follow-up until early adulthood in autosomal dominant, complex SPG30 with a novel KIF1A variant: a case report

**DOI:** 10.1186/s13052-019-0752-5

**Published:** 2019-12-03

**Authors:** Carlotta Spagnoli, Susanna Rizzi, Grazia Gabriella Salerno, Daniele Frattini, Carlo Fusco

**Affiliations:** 1 Neuropsichiatria Infantile, Presidio Ospedaliero Provinciale S. Maria Nuova, Azienda USL-IRCCS di Reggio Emilia, Reggio Emilia, Italy; 2 SC Neuropsichiatria Infantile Laboratorio di Neurofisiologia dell’Età Evolutiva. Presidio Ospedaliero Provinciale S. Maria Nuova, Azienda USL-IRCCS di Reggio Emilia, Reggio Emilia, Italy

**Keywords:** Cerebellar atrophy, Hereditary spastic paraplegia, SPG30, *KIF1A*

## Abstract

**Background:**

Pathogenic variants in *KIF1A* (kinesin family member 1A) gene have been associated with hereditary spastic paraplegia (HSP) type 30 (SPG30), encopassing autosomal dominant and recessive, pure and complicated forms.

**Case presentation:**

We report the long-term follow-up of a 19 years-old boy first evaluated at 18 months of age because of toe walking and unstable gait with frequent falls. He developed speech delay, mild intellectual disability, a slowly progressive pyramidal syndrome, microcephaly, bilateral optic subatrophy and a sensory axonal polyneuropathy. Brain MRI showed cerebellar atrophy, stable along serial evaluations (last performed at 18 years of age). Targeted NGS sequencing disclosed the de novo c.914C > T missense, likely pathogenic variant on *KIF1A* gene.

**Conclusions:**

We report on a previously unpublished de novo heterozygous likely pathogenic *KIF1A* variant associated with slowly progressive complicated SPG30 and stable cerebellar atrophy on long-term follow-up, adding to current knowledge on this HSP subtype.

## Background

Hereditary spastic paraplegias (HSP) are a group of clinically and genetically heterogeneous neurodegenerative disorders featuring progressive loss of corticospinal motor tract function, resulting in spasticity predominantly in the lower limbs, which can be isolated (pure forms) or associated with additional neurological or non-neurological features (complex forms) [1, 2].

Pathogenic variants in *KIF1A* were first associated with two neurodegenerative diseases in 2011: hereditary sensory and autonomic neuropathy (HSAN) type 2 [3] and a form of AR hereditary spastic paraparesis (HSP) [4]. One year later, it was demonstrated that *KIF1A* pathogenic variants cause SPG30 [5], and in the following years, both AR [6] and AD [7, 8], pure and complicated [7, 9] forms of HSP were described in association with *KIF1A* pathogenic variants. A third phenotype (mental retardation, autosomal dominant 9) (OMIM #614255), initially thought to represent a distinct disease, has been later demonstrated to be also caused by *KIF1A* pathogenic variants.

Description of long-term follow-up data on patients with rare neurogenetic conditions can be crucial to increase our knowledge on the natural history of the disease and to correctly disentangle it from phenotypic heterogeneity [10].

We present the clinical, neuroimaging and genetic data on a patient recently diagnosed with SPG30 with long-term neurologic follow-up, ranging from infancy to early adulthood.

## Case presentation

A male patient, now aged 19 years, was born at term after uneventful pregnancy and delivery (Apgar scores: 9, 9, birth weight 3680 g), from healthy non-consanguineous Caucasian parents. He has an older sister reportedly toe walking in the first 2 years of life. His early developmental milestones were normally achieved (independent sitting at 6 months, first words at 12 months). He made his first steps at 14 months, however, concerns arose regarding toe walking and unstable gait with frequent falls, and he was first referred to child neurology evaluation at 18 months of age. Pyramidal signs at his lower limbs were detected. Subsequently, he developed speech delay, learning difficulties, and mild intellectual disability. During follow-up, microcephaly, bilateral optic subatrophy, hyperlordosis and asymmetric equino-valgo-pronated feet also became evident. Moreover, his spastic paraparesis has shown a slowly progressive course, with increasing difficulties in independent walking and a worsening in his hypertonus in the lower limbs. No quantitative scale was administered during follow-up.

At the last follow-up, he is slightly microcephalic (OFC = 53 cm, < − 2 SD). He shows toe walking with a broad-based gait and valgo-pronated feet.

**Fig. 1:**
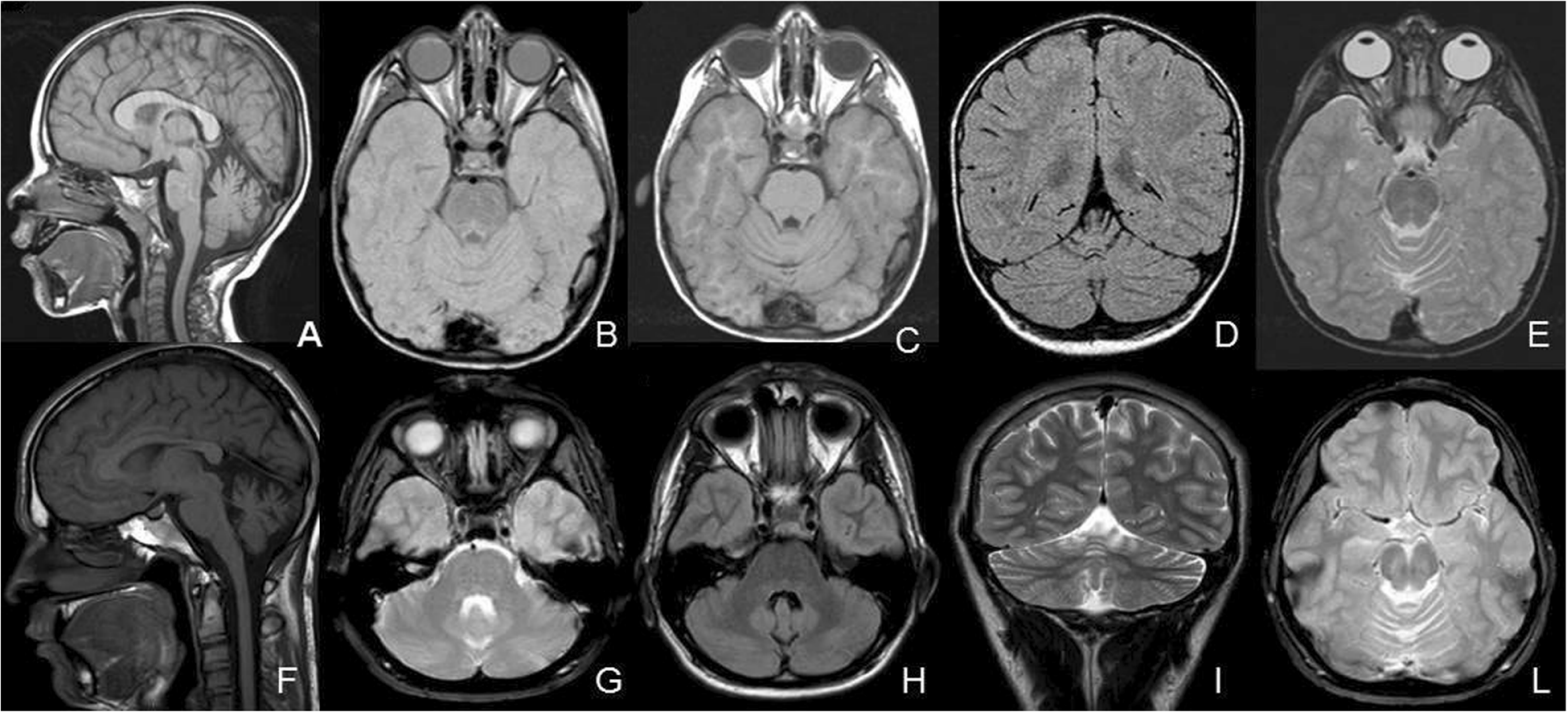
Brain MRI at 4 (first row) and18 (second row) years of age, showing stable superior vermis atrophy and reduced volume of the optic nerves. **a** and **f**: sagittal T1 sequences, **b** and **g**: axial T2 sequences, **c** and **h**: longitudinal FLAIR sequences, **d**: coronal FLAIR and **i**: coronal T2 sequences, **e** and **l**: longitudinal T2 sequences

During this long follow-up, he underwent extended diagnostic investigations. Brain MRI, first performed at 18 months of age, documented superior vermis atrophy, and reduced volume of the optic nerves and optic chiasm, with no significant progression (last examined at 18 years of age) (Fig. 1).

Somatosensory evoked potentials at 12 years of age disclosed asymmetrically increased latencies of the central sensory conduction velocity. Serial ENG/EMG tests initially showed normal results, but at 14 years of age a sensory axonal polyneuropathy was diagnosed, showing evolution towards sensory-motor involvement at all four limbs (aged 18 years).

Negative neurometabolic and neuromuscular investigations include alpha-fetoprotein, amino acids, beta-exosaminidase, muscle biopsy with immuno-hystochemistry, and respiratory chain enzimes activity. Neurogenetically-wise, direct sequencing of POLG, Twinkle, PLA2G6, OPA1, OPA3, SPG20, and SCA5 retrieved negative results, while two known polymorphisms were detected on the exons 11 and 15 of the SPG7 gene. Array-CGH detected two partial duplications, both of maternal origin: arr3q29(197,728,559-197,840,339) x3mat (including the *LMLN* gene) and arr4q22.1(93,202,223-93,359,983) x3mat (including the *GRID2* gene).

A targeted NGS panel for hereditary spastic paraplegia and motoneuronal disorders disclosed the heterozygous candidate variant 241,715,312 NM_001244008.1 c.914C > T (NP_001230937.1 p.Pro305Leu) on *KIF1A* gene, arisen de novo.

## Discussion and conclusions

The variant we identified is a previously unpublished in the literature, missense, non-tolerated variant, absent in controls. It has been previously reported in ClinVar in four cases, in two in association with mental retardation, autosomal dominant 9 (OMIM #614255), as a de novo variant, interepreted as likely pathogenic in the first patient and as pathogenic in the second one. Of note, the third and fourth cases report it as pathogenic, and in one the associated phenotype is characterized by developmental delay, cerebellar atrophy, ataxia, and eye movement abnormalities (patient tested at GeneDx). The variant is located inside the motor domain of kinesin 1A, in the N terminus of the protein (aa 1–365), where the vast majority of pathogenic variants associated with both AR and AD SPG30 are clustered. This domain is necessary for kinesin 1A movement along neurites and for binding with ATP and microtubules [11]. The p.Pro305Leu variant is a semi-conservative amino acid substitution, which may impact on secondary protein structure. This substitution occurs at a position which is conserved across species, and in silico analysis predicts it as probably damaging.

The variant is classified as likely pathogenic according to ACMG criteria [12]: PM1: Located in a mutational hot spot and/or critical and well-established functional domain (e.g., active site of an enzyme) without benign variation; PM2: Absent from controls (or at extremely low frequency if recessive) in Exome Sequencing Project, 1000 Genomes Project, or Exome Aggregation Consortium; PP3: Multiple lines of computational evidence support a deleterious effect on the gene or gene product (conservation, evolutionary, splicing impact, etc.); PP5: Reputable source recently reports variant as pathogenic, but the evidence is not available to the laboratory to perform an independent evaluation (InterVar, last examined 10th October 2019).

The clinical picture associated with complicated forms of SPG30 typically features intellectual disability, along with cerebellar atrophy and spastic paraplegia, with very wide range of onset. In some cases, optic nerve atrophy, thinning of the corpus callosum, periventricular white matter lesions, epilepsy and microcephaly (in two literature patients as well as the present one) [9], have also been described. The broad spectrum of observed clinical features likely reflects the ubiquitous expression of KIF1A in the nervous system, with a key role in anterograde transport of synaptic vescicles along axons [13]. Here we report a case of complicated, AD-SPG30 associated with a novel candidate missense variant located in the motor domain and characterized by the typical clinical phenotype associating spasticity, intellectual disability, cerebellar involvement and neuropathy [6].

Clinically, in contrast with the first descriptions of heterozygous cases, initially reported to be associated to both younger age at onset and more severe phenotypes [14], our patient has shown a slow progression of his pyramidal syndrome in his first 19 years of life, while cerebellar atrophy has remained unchanged up to 18 years (Fig. 1). Cerebellar atrophy is an almost constant finding in complicated forms of SPG30, but the majority of studies lack data on its temporal evolution. Hotchkiss and colleagues described two unrelated patients with progressive cerebellar atrophy demonstrated on sequential brain MRI’s performed between 11 months and 12.5 years in the first and between 6 months and 6 years in the second one. Interestingly, these patients had normal cerebellar size when first imaged in infancy [15]. To the best of our knowledge, the oldest published patient with serial brain MRI’s is a 27 years old female patient with no progression of cerebellar atrophy between 11 and 26 years of age [14]. In our case, there is no progression in cerebellar atrophy in the long-term follow-up from infancy to early adulthood, even though the patient harbours a variant in the heterozygous state.

In summary, with long-term follow-up, this case adds to current knowledge on phenotypic and genotypic variability in complex AD SPG30.

## Data Availability

Data and materials will be made available upon request.

## Abbreviations

AD: Autosomal dominant
AR: Autosomal recessive
Array-CGH: array-comparative genomic hybridization
ATP: Adenyl-triphosphate
EMG: Electromyography
ENG: Electroneurography
g: grams
GRID2: Glutamate receptor ionotropic delta 2
HSAN: Hereditary sensory and autonomic neuropathy
HSP: Hereditary spastic paraplegia
KIF1A: Kinesin family member 1A
LMLN: Leishmanolysin-Like (Metallopeptidase M8 Family)
MRI: Magnetic resonance imaging
NGS: Next generation sequencing
NM: mRNA in Reference Sequence
NP: Protein in Reference Sequence
OFC: Occipito-frontal circumference
OMIM: Online Mendelian Inheritance in Man
OPA1: Optic atrophy 1
OPA3: Optic atrophy 3
PLA2G6: Phospholipase A2 group VI
POLG: Polymerase gamma
SCA5: Spinocerebellar ataxia type 5
SD: Standard deviation
SPG20: Spastic paraplegia type 20
SPG30: Spastic paraplegia type 30
SPG7: Spastic paraplegia type 7
